# Possible Antagonism between *Cladosporium cladosporioides* and *Microcystis aeruginosa* in a Freshwater Lake during Bloom Seasons

**DOI:** 10.3390/life12050742

**Published:** 2022-05-17

**Authors:** Larry Wymer, Stephen Vesper, Ian Struewing, Joel Allen, Jingrang Lu

**Affiliations:** Center Environmental Measurement and Modeling, United States Environmental Protection Agency, 26 W. Martin Luther King Drive, Cincinnati, OH 45268, USA; wymer.larry@epa.gov (L.W.); struewing.ian@epa.gov (I.S.); allen.joel@epa.gov (J.A.); lu.jingrang@epa.gov (J.L.)

**Keywords:** *Cladosporium cladosporioides*, *Microcystis aeruginosa*, cyanobacteria, bloom, microcystin, antagonism

## Abstract

To ensure drinking-water safety, it is necessary to understand the factors that regulate harmful cyanobacterial blooms (HCBs) and the toxins they produce. One controlling factor might be any relationship between fungi and the cyanobacteria. To test this possibility, water samples were obtained from Harsha Lake in southwestern Ohio during the 2015, 2016, and 2017 bloom seasons, i.e., late May through September. In each water sample, the concentration of the filamentous fungus *Cladosporium cladosporioides* was determined by quantitative PCR (qPCR) assay, and *Microcystis aeruginosa* microcystin-gene transcript copy number (McyG TCN) was quantified by reverse-transcriptase qPCR (RT-qPCR) analyses. The results showed that during each bloom season, the *C. cladosporioides* concentration and McyG TCN appeared to be interrelated. Therefore, *C. cladosporioides* concentrations were statistically evaluated via regression on McyG TCN in the water samples for lag times of 1 to 7 days. The regression equation developed to model the relationship demonstrated that a change in the *C. cladosporioides* concentration resulted in an opposing change in McyG TCN over an approximately 7-day interval. Although the interaction between *C. cladosporioides* and McyG TCN was observed in each bloom season, the magnitude of each component varied yearly. To better understand this possible interaction, outdoor *Cladosporium* spore-count data for the Harsha Lake region were obtained for late May through September of each year from the South West Ohio Air Quality Agency. The average *Cladosporium* spore count in the outdoor air samples was significantly greater in 2016 than in either 2015 or 2017, and the *M. aeruginosa* McyG TCN was significantly lower in Harsha Lake water samples in 2016 compared to 2015 or 2017. These results suggest that there might be a “balanced antagonism” between *C. cladosporioides* and *M. aeruginosa* during the bloom season.

## 1. Introduction

To ensure drinking-water safety, it is necessary to understand the factors that regulate harmful cyanobacterial blooms (HCBs) and the toxins they produce. *Microcystis aeruginosa* is the cyanobacterial species most often identified in freshwater blooms [1,2,3,4,5]. These blooms become harmful because they produce a class of cyanotoxins called microcystins [6,7]. Our attention was drawn to the possible role of fungi in cyanobacterial bloom regulation by the work of Cooley et al. [8].

Cooley et al. [8] reported that the filamentous fungus *Cladosporium* *cladosporioides* (Fresenius) de Vries grew in close association with the green marine alga *Pseudendoclonium submarinum.* Under most circumstances, the association was limited to the fungal hyphae infiltrating into the intercellular areas of the algal filaments, not penetrating the algal cell walls. However, under conditions of high algal density, such as in culture, the alga declined and became necrotic. *Cladosporium cladosporioides* appeared to become pathogenic to the alga resulting in a “balanced antagonism” between the fungus and the alga.

*Cladosporium cladosporioides* is the most numerous fungus in outdoor air, worldwide [9,10]. Because *C. cladosporioides* occurs in large numbers in the air, it is deposited in large numbers in aquatic environments. Our hypothesis is that there might be an interactive relationship between *C. cladosporioides* and *M. aeruginosa* during a bloom. We examined this possibility during the three bloom seasons, 2015–2017, in a freshwater lake in Ohio.

## 2. Materials and Methods

### 2.1. Study Site and Water Sample Collection and Analyses

The William H. Harsha Lake (hereafter, Harsha Lake) is an engineered reservoir that was developed in 1978, and its location and characteristics have been described in detail [11]. Briefly, Harsha Lake was created on a branch of the Little Miami River in Clermont County, Ohio, about 40 km east of Cincinnati. Harsha Lake has an average depth of 13.1 m, covers an area of 8.7 km^2^, and drains from a watershed of 890 km^2^, with 64% of the land used for agriculture and 26% comprised of forest cover. The development of HCBs in Harsha Lake has been studied by our team since 2015 [11,12,13,14]. In this study, the water samples were collected from late May through September in 2015, 2016, and 2017.

One-liter water samples were collected near a marker buoy installed in Harsha Lake, as previously described [13]. The samples were collected at a depth of approximately 15 to 30 cm (6–12 inches) in five Polyethylene Terephthalate Glycol (PTEG) bottles (Thermo-Fischer, Waltham, MA, USA). Samples were obtained every one to three days during the peak of the bloom and every four to five days later in the season. A 100–200 mL aliquot of each sample was filtered through a Durapore™ polyvinylidene fluoride (PVDF) filter (0.45 μm, MilliPore, Foster City, CA, USA). The filter was then recovered and inserted into a 1.5 mL microfuge tube containing 600 μL of the “RLT plus buffer” (QIAGEN, Valencia, CA, USA) and stored at −80 °C. These filters were used for the quantification of *Cladosporium cladosporioides* by qPCR assay and McyG TCN using reverse-transcriptase qPCR (RT-qPCR) analysis.

Each filter was disrupted and lysed using a Mini-Beadbeater-16 (BioSpec Products, Inc., Bartlesville, OK, USA) with two pulses for 30 s each. The mixture was then centrifuged at 10,000× *g* for 3 min. The supernatant was then carefully transferred to a new sterile tube and the extracted DNA purified using the “AllPrep DNA” kit (QIAGEN, Valencia, CA, USA), per the manufacturer’s instructions. DNA concentrations were estimated with a Nanodrop ND-1000 Spectrophotometer (NanoDrop Technologies, Inc., Wilmington, DE, USA). The extracts were stored at −80 °C until qPCR and RT-qPCR analyses were performed.

### 2.2. Quantification of Microcystis aeruginosa Microcystin-Gene Transcript Copy Number (McyG TCN) in Harsha Lake Water Samples

The *Microcystis aeruginosa* microcystin-gene transcript copy number (McyG TCN) was measured by RT-qPCR, as previously described [14]. First, the DNA in the samples was removed from the total extracts using TURBO DNA-free™ (Life Technologies Co., Carlsbad, CA, USA). Then, the RNA was transcribed to cDNA using the High-Capacity cDNA reverse transcription kit (Life Technologies Co.), per the manufacturer’s instructions. After transcription, 2 μL of template cDNA was added to 2 × qPCR SYBR Green Master Mix (Life Technologies Co.) and 0.2 μM primers (Forward Primer: CAACCCAACAGGTTCTTAAAGC; Reverse Primer: TGAGGCAAGGTTTCCTCTTG) to make a 20 μL assay mix for each qPCR reaction. Pre-amplification conditions consisted of a 50 °C treatment for 2 min with Uracil-N-Glycosylase (UNG) to prevent carryover contamination, followed by 95 °C for 10 min to denature the cDNA. Amplification was performed on a QuantStudio™ 6 Flex system (Life Technologies) using the protocol of 40 cycles at 95 °C for 15 s and at 60 °C for 30 s, with an extension at 72 °C for 30 s and a final hold-step at 72 °C for 5 min.

Each sample was run in replicate and each qPCR plate contained a duplicate six-point (10^1^–10^6^ copies) standard curve [15]. The standard curve was based on dilutions of *Microcystis aeruginosa* genomic DNA starting at 5 ng/µL. The McyG TCN was calculated by multiplying by Avogadro’s number and dividing by the genome size. The McyG TCN for each sample was calculated using an equation generated from the average of the standard curves in each plate. The final McyG TCN was calculated by multiplying by the dilution factor and a water-filtering factor to present data as McyG TCN per mL of filtered lake water.

### 2.3. Quantification of Cladosporium cladosporioides in Harsha Lake Water Samples

*Cladosporium cladosporioides* was quantified using a qPCR assay with Forward Primer: CATTACAAGTGACCCCGGTCTAAC; Reverse Primer: CCCCGGAGGCAACAGAG; and Probe: CCGGGATGTTCATAACCCTTTGTTGTCC [16]. The standard qPCR assay contained 12.5 µL of “Universal Master Mix” (Applied Biosystems Inc., Foster City, CA, USA), 1 µL of a mixture of forward and reverse primers at 25 µM each, 2.5 µL of a 400 nM TaqMan probe (Applied Biosystems Inc.), 2.5 µL of 2 mg/mL fraction V bovine serum albumin (Sigma Chemical, St. Louis, MO, USA) and 2.5 µL of DNA-free water (Cepheid, Sunnyvale, CA, USA). To this mix we added 5 µL of the DNA extract from the sample. Reactions were performed with thermal cycling conditions consisting of 2 min at 50 °C, 10 min at 95 °C, followed by 40 cycles of 15 s at 95 °C for template denaturation and 1 min at 60 °C for probe and primer annealing and primer extension. The cycle threshold (Ct) determinations were automatically performed by the Roche 480 instrument (Roche Inc., Penzberg, Germany) using default parameters. The concentrations of *C. cladosporioides* were expressed as cell equivalents per ml of water sample (CE/mL) based on the comparison to a standard curve [17].

### 2.4. Cladosporium Spore Counts in Air Samples in the Region of Ohio That Includes Harsha Lake

Outdoor-spore counts for *Cladosporium* in the Harsha Lake region were obtained from the South West Ohio Air Quality Agency (SWOAQA), the local agency responsible for air quality measurements for the Greater Cincinnati region (http://www.southwestohioair.org/local_air_quality/pollen_and_mold (accessed on 17 June 2019)). The SWOAQA collected samples using a Rotorod^®®^ sampler (QuintilesIMS, Durham, NC, USA) and quantified the number of mold spores per m^3^ of air by microscopic observation. Samples were not obtained on weekends or holidays.

### 2.5. Statistical Analysis

Changes in McyG TCN (C_McyG_) over a period of from 1 to 7 days were evaluated as a function of initial (“day 0”) C_McyG_ and the concentration of *C. cladosporioides* relative to McyG, i.e., C_clad_/C_McyG_, on day 0. The log concentrations were used in a regression model, giving:

log(C_McyG,N_/C_McyG,0_) = a + b·log(C_McyG,0_) + c·log(C_clad,0_/C_McyG,0_) (1)

Here, the subscript 0 refers to concentration on day 1 and N refers to concentration after a period of a selected number of days. The estimated coefficients of the regression, which is in the form of a time series, are denoted by a, b, c.

The value for N was selected by estimating this equation for values of N ranging from 1 to 7 days later. The “optimal” N was chosen as the value resulting in the highest partial correlation of log(C_clad,0_/C_McyG,0_) among the 7 possible equations, given that the effect of *C. cladosporioides* is of primary interest in this analysis. The results for 2016 and 2017 were incorporated as “dummy” variables (i.e., 0, 1) to test for differences overall and with respect to slopes, relative to the arbitrarily selected “base” year, 2015.

One-way analysis of variance (ANOVA) was used to compare the concentrations of *C. cladosporioides* or McyG TCN in the water samples over the three bloom seasons. ANOVA was also used to compare *Cladosporium* spore counts in the outdoor air during late May through September each year. All analyses were performed using SAS version 9 (SAS Institute, Inc., Cary, NC, USA).

## 3. Results

**Figure 1:**
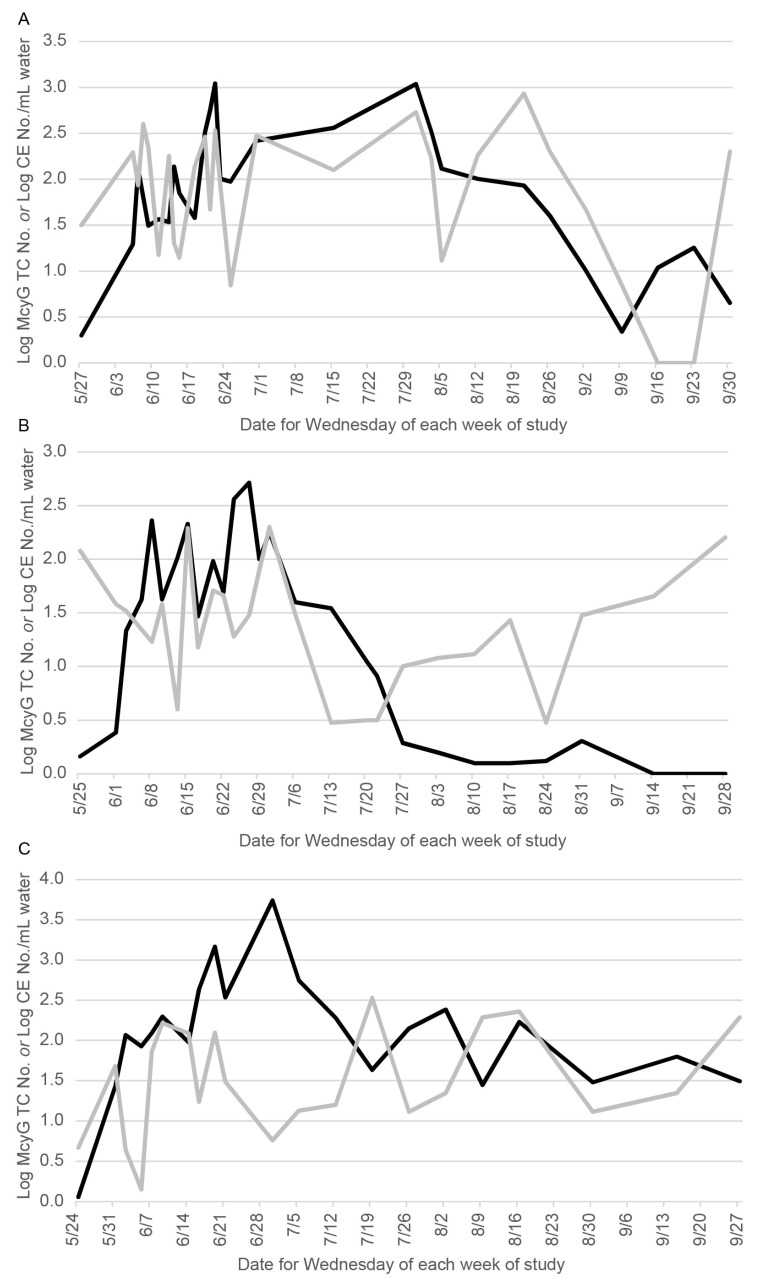
The log of the McyG transcript copy number (Log McyG TC No.) per mL water (black line) and the log of the *Cladosporium cladosporioides* cell equivalents number (Log CE No.) per ml of water (gray line) shown on the same days in 2015 (**A**), 2016 (**B**) or 2017 (**C**).

The McyG TCN began to be measurable in late May in each bloom season (Figure 1A–C), but even before late May, *C. cladosporioides* cells were already measurable in the water at concentrations of about 5 to 100 cells per mL water, depending on the year. Throughout the bloom season, as McyG TCN increased, it appeared that the concentrations of *C. cladosporioides* decreased and then vice versa. Similar “up-and-down” interactions seemed to have occurred in each bloom season. The regression model of this relationship, based on a 7-day time lag (*n* = 7) between samples in Equation (1) above, is given by:

log(C_McyG,7_/C_McyG,0_) = 1.4 − 0.68·log(C_McyG,0_) − 0.55·log(C_clad,0_/C_McyG,0_) (2)

The statistical significance was *p* < 0.0001 for each coefficient and the overall R^2^ was 0.67. Per our selection criteria, the 7-day lag time resulted in the highest partial correlation to the *C. cladosporioides* effect (0.53) vs. 1-to-6-day lags (range = 0.28–0.50). It was noted that *C. cladosporioides* effects were significantly negative for any other possible lag time of 1 to 6 at *p* < 0.01, and overall R^2^ values were in the range 0.46–0.69. This lends support to consistency among results regardless of lag times between samples.

The first two terms of Equation (2) imply the logical conclusion that the higher the initial concentration of McyG TCN, the less it can be expected to increase. The term log(C_clad,0_/C_McyG,0_) indicates that as the concentration of *C. cladosporioides* relative to McyG TCN increases, there appears to be an inhibitory effect on potential temporal increase in McyG TCN. This does not occur if only C_clad,_ is used rather than its ratio to the corresponding C_McyG_, in which case the coefficient is not statistically significant (*p* = 0.51). Thus, *C. cladosporioides* concentrations appear to have a significant negative relationship with the 7-day change in McyG TCN based on the *C. cladosporioides*’ concentration relative to that of McyG TCN.

Therefore, the regression implies that a change in the *C. cladosporioides* concentrations is accompanied by an opposing change in McyG TCN over an approximately one-week interval. This relationship was consistently found in each year, as demonstrated by the fact that the intercept and coefficients did not differ significantly (*p* = 0.62) during each bloom season.

**Figure 2:**
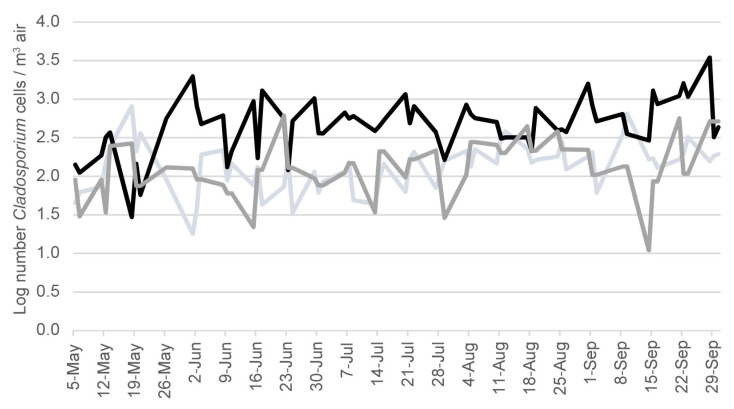
Log of the number of *Cladosporium* spores in outdoor air samples in 2015 (light gray), 2016 (gray) and 2017 (black) in the Harsha Lake region, as reported by the South West Ohio Air Quality Agency.

Although the relationship between *C. cladosporioides* concentrations and McyG TCN were seen in each bloom season, the magnitude varied each year. For example, the mean *C. cladosporioides* concentration and the mean McyG TCN in the water samples were lower in 2016 compared to either 2015 or 2017 (Table 1). In contrast, the mean *Cladosporium* spore concentration in the outdoor air samples was significantly greater in the 2016 bloom season compared to 2015 or 2017 (Figure 2).

## 4. Discussion

The regression equation developed to model the interaction between *C. cladosporioides* and *M. aeruginosa* McyG TCN suggests that a change in the *C. cladosporioides* concentrations was associated with an opposing change in McyG TCN over an approximately one-week interval. This possible association does not prove that there is a causal interaction. We recognize that this quantitative relationship could simply be a coincidence within the complex dynamics of an algal bloom, which is affected by many other factors, including the nutrients nitrogen (N) and phosphorous (P), rainfall or other water inputs, and many types of organisms. The next step will be a controlled experiment limited to the addition of *C. cladosporioides* and *M. aeruginosa* at varied concentrations and in well-defined nutrient and environmental conditions.

Of the factors to consider in HCBs development, the concentrations of N and P are critical for HCB development, e.g., in [18], and measuring these nutrients is essential for modeling and predicting HCB development [13,19]. However, it is unclear if nutrient load affects the interaction between *C. cladosporioides* and *M. aeruginosa*. Previously, we demonstrated that heterotrophic bacteria could compete with *M. aeruginosa* for N and P if an additional carbon source was provided [11].

Models are increasingly being created and tested to clarify the relevant factors in HCB development, although the role of the interaction between *C. cladosporioides* and *M. aeruginosa* has not been considered. Su et al. [20] identified some factors that affected the occurrence of HCBs in the Xiangxi River, a tributary of the Three Gorges Reservoir, China (2017–2018), for the dry season (October to mid-April) and wet season (April to September). To evaluate the most relevant factors in HCB development, they utilized maximal information coefficient analysis combined with a time lag strategy. For the 2017–2018 bloom season, they found that water temperature was an important factor in HCB development. Total nitrogen, even more so than phosphorous, affected HCB in both the wet and dry seasons. However, depending on the season, the operational control of water inflow and outflow into reservoir was found to be the most significant factor in HCB development.

Gathering sufficient temporal data was found to be critical to understanding HCB ecology [21]. However, in some studies, water sampling only occurred on a weekly or even monthly basis. Any dynamic interactions might have been missed. Kim et al. [21] developed HCB predictive models that include meteorological, hydrological, environmental, and biological factors for the Nakdong and Yeongsan Rivers in South Korea. The factors evaluated appeared to be most useful for modeling when considered in the context of monitoring 1 to 7 days prior to forecasting [20]. This is consistent with the 7-day lag in the possible interaction between *C. cladosporioides* and *M. aeruginosa* that we observed in our model.

*C. cladosporioides* is only one of many aquatic organisms that could affect *M. aeruginosa* HCB development. Bacteria, viruses, fish and zooplankton can infect, lyse, or consume toxin-producing cyanobacteria, including *M. aeruginosa* [22]. Fungi have been shown to have “algicidal” activity against cyanobacteria [23]. If there is interaction between *M. aeruginosa* and *C. cladosporioides*, it might be created by the release of competing toxins or other metabolites.

Microcystins are protein phosphatase inhibitors for eucaryotes, e.g., *C. cladosporioides,* causing hyperphosphorylation of cytoskeleton proteins resulting in toxicity to eukaryotic cells [24,25]. On the other hand, *Cladosporium* species produce toxins such as perylenequinones and enzymes, which can disrupt cell membranes [26]. Therefore, it might be reasonably hypothesized that *C. cladosporioides* and *M. aeruginosa* form some type of antagonistic relationship because of these chemicals.

*Cladosporium cladosporioides* is known to form many types of relationships with both aquatic and terrestrial plants and algae that include endophytic [27], saprophytic [28], weakly parasitic [29], and pathogenic states [30]. Cooley et al. [8] described the relationship between *C. cladosporioides* and the marine alga *Pseudendoclonium submarinum* as a “balanced antagonism,” and the results presented here suggest that there might be a similar “balanced antagonism” between *C. cladosporioides* and *Microcystis aeruginosa*. If there is a “balanced antagonism”, it may start very early in the bloom season.

Early each summer, *C. cladosporioides* cells were already present in the lake water even before any evidence of McyG transcript copies from *M. aeruginosa* was detected. It may be that the outdoor air “seeds” the water with *C. cladosporioides*, even before the bloom begins. If so, seasonal or yearly differences in *Cladosporium* in the outdoor air may affect *M. aeruginosa* bloom development. *Cladosporium* is a phylloplane fungus, and its population can vary depending on foliage development each year [9,10].

The outdoor air concentration of *Cladosporium* was 5 to 10 times greater in 2016 than in either 2015 or 2017. In 2016, *M. aeruginosa* McyG TCN were lower than in 2015 or 2017 in the water samples from Harsha Lake. One possibility is that the higher outdoor air concentrations of *Cladosporium* in 2016 suppressed the *M. aeruginosa*. More surveillance studies will be needed to test this possibility.

There are limitations to our findings, since only one lake was studied, and only during three bloom seasons. Moreover, samples were collected on an irregular basis, which limited our ability to accurately time any possible interactions. In addition, the SWOAQA does not speciate the fungal spores it counts. Therefore, we cannot be certain that the *Cladosporium* spores counted in in the air samples were all *C. cladosporioides.* However, since *C. cladosporioides* is the dominant species of mold in outdoor air, worldwide [9], it seems likely that a large percentage of these *Cladosporium* spores were of this species.

## 5. Conclusions

*Cladosporium cladosporioides* and *M. aeruginosa* appear to develop a “balanced antagonism” during HCB development.

## Figures and Tables

**Table 1:** Shown are the number of samples collected for each year studied. Then, the mean and standard deviation (SD) of the McyG transcript copy number (Log McyG TCN) per ml of water, the mean and standard deviations (SD) of the concentration of *Cladosporium cladosporioides* (C. clad.) measured by qPCR as log cell equivalents (CE) per ml of water (Log CE. C. clad. Water), and the log mean and SD of the *Cladosporium* number of spores per m^3^ air (Log No. Clados. Air) are given.

Study	Sample	Log McyG TCN	SD	Sample	Log CE. C. Clad. Water	SD	Sample	Log No. Clados. Air	SD
Year	Number	Mean	+/−	Number	Mean	+/−	Number	Mean	+/−
2015	(*n* = 28)	2.00	0.72	(*n* = 28)	1.90 ^b^	0.77	(*n* = 53)	2.11	0.29
2016	(*n* = 27)	1.31 ^a^	0.91	(*n* = 27)	1.33	0.54	(*n* = 53)	2.74 ^c^	0.29
2017	(*n* = 21)	2.07	0.74	(*n* = 21)	1.50	0.66	(*n* = 53)	2.14	0.35

^a^ Mean for 2016 is significantly lower than 2015 (*p* = 0.001) and 2017 (*p* = 0.002). ^b^ Mean for 2015 is significantly higher than 2016 (*p* < 0.001) and 2017 (*p* = 0.015). ^c^ Mean for 2016 is significantly higher than 2015 and 2017 (*p* < 0.001).

## Data Availability

All data will be available at the NIH-PMC website.

## References

[1] Carmichael W.W. (2001). Health effects of toxin-producing cyanobacteria: The CyanoHABS. Hum. Ecol. Risk Assess. Int. J..

[2] Leflaive J., Ten-Hage L.O.Ï.C. (2007). Algal and cyanobacterial secondary metabolites in freshwaters: A comparison of allelopathic compounds and toxins. Freshw. Biol..

[3] Pearson L., Mihali T., Moffitt M., Kellmann R., Neilan B. (2010). On the chemistry, toxicology and genetics of the cyanobacterial toxins, microcystin, nodularin, saxitoxin and cylindrospermopsin. Mar. Drugs.

[4] Wiegand C., Pflugmacher S. (2005). Ecotoxicological effects of selected cyanobacterial secondary metabolites: A short review. Toxicol. Appl. Pharmacol..

[5] Zurawell R.W., Chen H., Burke J.M., Prepas E.E. (2005). Hepatotoxic cyanobacteria: A review of the biological importance of microcystins in freshwater environments. J. Toxicol. Environ. Health Part B.

[6] Schreidah C.M., Ratnayake K., Senarath K., Karunarathne A. (2020). Microcystins: Biogenesis, toxicity, analysis, and control. Chem. Res. Toxicol..

[7] Omidi A., Pflugmacher S., Kaplan A., Kim Y.J., Esterhuizen M. (2021). Reviewing interspecies interactions as a driving force affecting the community structure in lakes via cyanotoxins. Microorganisms.

[8] Cooley D.R., Mullins R.F., Bradley P.M., Wilce R.T. (2011). Culture of the upper littoral zone marine alga *Pseudendoclonium submarinum* induces pathogenic interaction with the fungus *Cladosporium cladosporioides*. Phycologia.

[9] Miller J.D. (1992). Fungi as contaminants in indoor air. Atmos. Environ..

[10] Nix-Stohr S., Moshe R., Dighton J. (2008). Effects of propagule density and survival strategies on establishment and growth: Further investigations in the phylloplane fungal model system. Micro. Ecol..

[11] Vesper S., Sienkiewicz N., Struewing I., Linz D., Lu J. (2022). Prophylactic addition of glucose suppresses cyanobacterial abundance in lake water. Life.

[12] Chen K., Allen J., Lu J. (2017). Community structures of phytoplankton with emphasis on toxic cyanobacteria in an Ohio inland lake during bloom season. J. Water Resour. Prot..

[13] Lu J., Zhu B., Struewing I., Xu N., Duan S. (2019). Nitrogen-phosphorus-associated metabolic activities during the development of a cyanobacterial bloom revealed by metatranscriptomics. Sci. Rep..

[14] Zhu B., Cao H., Li G., Du W., Xu G., Domingo J.S., Gu H., Xu N., Duan S., Lu J. (2019). Biodiversity and dynamics of cyanobacterial communities during blooms in temperate lake (Harsha Lake, Ohio, USA). Harmful Algae.

[15] Church M.J., Short C.M., Jenkins B.D., Karl D.M., Zehr J.P. (2005). Temporal patterns of nitrogenase gene (nifH) expression in the oligotrophic North Pacific Ocean. Appl. Environ. Microbiol..

[16] Haugland R.A., Vesper S.J. (2002). Identification and Quantification of Specific Fungi and Bacteria. U.S. Patent.

[17] Haugland R.A., Varma M., Wymer L.J., Vesper S.J. (2004). Quantitative PCR of selected *Aspergillus*, *Penicillium* and *Paecilomyces* species. Syst. Appl. Microbiol..

[18] Barnard M.A., Chaffin J.D., Plaas H.E., Boyer G.L., Wei B., Wilhelm S.W., Rossignol K.L., Braddy J.S., Bullerjahn G.S., Bridgeman T.B. (2021). Roles of Nutrient Limitation on Western Lake Erie CyanoHAB Toxin Production. Toxins.

[19] Wang K., Mou X., Cao H., Struewing I., Allen J., Lu J. (2021). Co-occurring microorganisms regulate the succession of cyanobacterial harmful algal blooms. Environ. Pollut..

[20] Su Y., Hu M., Wang Y., Zhang H., He C., Wang Y., Wang D., Wu X., Zhuang Y., Hong S. (2022). Identifying key drivers of harmful algal blooms in a tributary of the Three Gorges Reservoir between different seasons: Causality based on data-driven methods. Environ. Pollut..

[21] Kim T., Shin J., Lee D., Kim Y., Na E., Park J.H., Lim C., Cha Y. (2022). Simultaneous feature engineering and interpretation: Forecasting harmful algal blooms using a deep learning approach. Water Res..

[22] Pal M., Yesankar P.J., Dwivedi A., Qureshi A. (2020). Biotic control of harmful algal blooms (HABs): A brief review. J. Environ. Manag..

[23] Han G., Feng X., Jia Y., Wang C., He X., Zhou Q., Tian X. (2011). Isolation and evaluation of terrestrial fungi with algicidal ability from Zijin Mountain, Nanjing, China. J. Microbiol..

[24] Pearson L.A., Hisbergues M., Börner T., Dittmann E., Neilan B.A. (2004). Inactivation of an ABC transporter gene, *mcy*H, results in loss of microcystin production in the cyanobacterium *Microcystis aeruginosa* PCC 7806. Appl. Environ. Microbiol..

[25] Runnegar M., Berndt N., Kong S.M., Lee E.Y., Zhang L. (1995). In vivo and in vitro binding of microcystin to protein phosphatases 1 and 2A. Biochem. Biophys. Res. Commun..

[26] Daub M.E., Ehrenshaft M. (2000). The photoactivated *Cercospora* toxin cercosporin: Contributions to plant disease and fundamental biology. Ann. Rev. Phyto..

[27] Schultz B., Boyle C. (2005). The endophytic continuum. Mycol. Res..

[28] Sadaka N., Ponge J.F. (2003). Fungal colonization of phyllosphere and litter of *Quercus rotundifolia* Lam. in a holm oak forest (High Atlas, Morocco). Biol. Fertil. Soils.

[29] O’Donnell J., Dickinson C.H. (1980). Pathogenicity of *Alternaria* and *Cladosporium* isolates on *Phaseolus*. Trans. Br. Mycol. Soc..

[30] Briceno E.X., Latorre B.A. (2008). Characterization of *Cladosporium* rot in grapevines, a problem of growing importance in Chile. Plant Dis..

